# Evaluation of the Immune Response of a Candidate Phage-Based Vaccine against *Rhipicephalus microplus* (Cattle Tick)

**DOI:** 10.3390/pharmaceutics13122018

**Published:** 2021-11-26

**Authors:** Alejandro González-Mora, Kenny Misael Calvillo-Rodríguez, Jesús Hernández-Pérez, Marco Rito-Palomares, Ana Carolina Martínez-Torres, Jorge Benavides

**Affiliations:** 1 Tecnologico de Monterrey, School of Engineering and Sciences, Campus Monterrey, Ave. Eugenio Garza Sada 2501, Monterrey 64849, N.L. CP, Mexico; alejandroglezm24@gmail.com (A.G.-M.); jhz.perez@tec.mx (J.H.-P.); 2 Laboratorio de Inmunología y Virología, Facultad de Ciencias Biológicas, Universidad Autónoma de Nuevo León, San Nicolás de los Garza 66455, N.L. CP, Mexico; kenny.misaelcr@gmail.com; 3 Tecnologico de Monterrey, Escuela de Medicina y Ciencias de la Salud, Ave. Morones Prieto 3000 Pte, Monterrey 64710, N.L. CP, Mexico; mrito@tec.mx

**Keywords:** phage-based vaccine, bacteriophage M13, phage display technology, cattle tick, *Rhipicephalus microplus*, bovine monocyte-derived dendritic cell, Bm86 protein, Sbm7462 peptide

## Abstract

Cattle tick (*Rhipicephalus microplus*) represents a severe problem causing substantial economic losses, estimated in billions of dollars annually. Currently, chemical acaricides represent the most widely used control method. However, several problems such as resistance have been described. Phage-based vaccines represent a fast and low-cost tool for antigen delivery. In this regard, the objective of the present work was to develop a candidate phage-based vaccine displaying a cattle tick antigen (Bm86-derived Sbm7462 antigen) on the surface of bacteriophage M13. Phage ELISA and dot blotting analysis confirmed the display of the antigen. Vaccine immunogenicity was evaluated using a bovine monocyte-derived dendritic cell-based ex vivo assay and a murine in vivo assay. The ex vivo model showed the maturation of dendritic cells after being pulsed with the phage-based vaccine. The humoral response was confirmed in the in vivo assay. These results demonstrated the capacity of the phage-based vaccine to induce both humoral and cellular immune-specific responses. Importantly, this is the first report describing a control method for cattle ticks using a candidate phage-based vaccine. Further studies to evaluate the immunogenicity in a bovine model are needed. The current approach represents a promising alternative to control cattle tick infestations.

## 1. Introduction

The cattle tick, *Rhipicephalus microplus*, is a blood-feeding ectoparasite that infests cattle in tropical and subtropical areas worldwide [1,2]. In the last decades, cattle tick has represented a serious problem in Mexico and worldwide, and it has been considered a primary veterinary challenge [3]. The cattle tick has an essential economic impact in the cattle industry estimated at billions of dollars annually [4,5].

Currently, several strategies intend to control the infestation of this parasite. The most used strategy involves the use of chemical acaricides with partially successful results [6]. In addition, the intensive use of such chemicals increases concerns regarding the presence of pesticide residues in cattle products (meat and milk) and the environment [6,7,8]. It has also been reported that the cost of the acaricide-based method is high due to labor and R&D expenses [9].

For the reasons described above, vaccines have been proposed as an alternative for controlling tick infestations. Nowadays, evidence suggests that anti-tick vaccines exert their action through the activation of both innate and adaptive immune responses [10]. In fact, the generation of specific antibodies that bind to antigens found in the digestive system of ticks such as the Bm86 antigen provokes intestinal cells damage, affecting their metabolism [11]. In this regard, the Sbm7462 antigen used here as the antigen model exerts a similar mechanism since it is derived from the Bm86 antigen. Thus, these immune responses allow the reduction of detached engorged ticks, reduction of eggs weight and reduction of fertility.

Although several types of vaccines have been evaluated, their low efficacy and high cost remain a severe problem that difficult the implementation this plague control method [10,12]. On the other hand, the production of recombinant antigens is expensive and technologically complex [13]. In order to overcome such limitations, the development of inexpensive bioprocesses has been proposed [14]. In that sense, recombinant bacteriophage technology represents a fast and low-cost tool for producing recombinant antigens [15]. Currently, two types of phage-based vaccines have gained attention: phage display and phage DNA vaccines. Phage display vaccines consist of the expression of antigens on the phage surface proteins to generate phages with immunogenic properties. Phage DNA vaccines are made of a eukaryotic expression cassette encoding antigens contained in a phage particle [16]. Other advantages of the phage display technology for its application on vaccine development include the adjuvant capacity of bacteriophages, high antigen stability, and high safety profile, among others [16,17,18,19]. Lack of studies regarding the impact of administration of phage preparations remains as one of the major limitations of both types of phage-based vaccines [20].

The objective of the present work was to investigate the use of the phage display technology to generate a candidate phage-based vaccine against cattle tick. Antigen Sbm7462, a peptide derived from cattle tick protein Bm86, was used as a model antigen. The immunogenicity of the phage-based vaccine was evaluated in a bovine monocyte-derived dendritic cell (MODC) based ex vivo assay as well as in a murine in vivo assay. Furthermore, this is the first study reporting the immunogenicity evaluation of a phage-based vaccine in a bovine cell-based ex vivo assay.

## 2. Materials and Methods

### 2.1. Biological Materials

*Escherichia coli* ER2738 and TG-1 strains (Lucigen Corporation, Middleton, WI, USA) were used to propagate VCSM13 helper phage and recombinant phages, respectively. Bacteriophage M13 (VCSM13 Interference-Resistant Helper phage) was purchased from Agilent Technologies (Santa Clara, CA, USA). Peripheral blood was collected from 6 healthy calves, *Bos taurus*, age from 3 to 9 months by a veterinarian service following the ethical recommendations of the Animal Ethical Committee (CEIBA).

### 2.2. Animals

In this case, 6- to 10-week-old male Balb/c mice were obtained from the animal house at the Universidad Autonoma de Nuevo Leon, Mexico. Animals were kept in cages in groups of six and maintained one week to become acclimated to the animal facility. Mice were housed under the following conditions: temperature 21 °C ± 3 °C, humidity 55% ± 10% and a 12-h light/dark cycle. Animals were fed with rodent maintenance food (LabDiet, St. Louis, MO, USA) and water ad libitum. Mice were randomly assigned for immunization. This study was approved by the Institutional Ethics Committee of Universidad Autónoma de Nuevo León, College of Biological Sciences in 30 October 2020 (CEIBA-2020-008).

### 2.3. Propagation of VCSM13 Helper Phage

VCSM13 helper phage was propagated in *Escherichia coli* strain ER2738 grown in a 1 L Erlenmeyer flask (25% working volume) using Super Broth (SB) medium (30 g L^−1^ tryptone enzymatic digest from casein, 20 g L^−1^ yeast extract, and 10 g L^−1^ MOPS) containing 70 µg mL^−1^ kanamycin. Culture conditions were 37 °C, 250 rpm, and 16 h (Shaking incubator 1585, VWR International, Radnor, PA, USA). The culture was then clarified by two centrifugation steps at 4 °C and 2500× *g* (Allegra 64R, C0650 rotor, Beckman, Brea, CA, USA) with a previous *E. coli* inactivation at 70 °C/20 min. The clarified supernatant obtained containing phage particles was stored at 4 °C and used as feedstock to propagate recombinant phages.

### 2.4. Construction of the Phagemid Vectors

A phagemid vector containing the sequences of the SBm7462^®^ Bm86 epitopes [21] (43 aa long) was constructed (pComb3x-antigen). The phagemid vector pComb3XTT that encoded a TT Fab against tetanus toxin (TT phage) was used as vector template as well as phage expression control (Addgene plasmid 63891) [22]. The antigen was expressed on the bacteriophage M13 surface by cloning the corresponding DNA fragments into pComb3xTT phagemid vector using the restriction sites of SfiI. All DNA sequences were optimized for their expression in *E. coli.* The genes were synthesized by Genscript (Piscataway, NJ, USA).

### 2.5. Preparation of Recombinant Phage Particles

The propagation of recombinant phages was performed according to the methodology proposed by Barbas et al., 2001 with some modifications [22]. Briefly, phagemid vector pComb3x-antigen was used to transform *E. coli* strain TG-1 by the electroporation method (2.5 kV). Transformants were selected for their resistance to carbenicillin. Positive colonies were used to prepare stock and stored at −80 °C for further experiments. An overnight culture of *E. coli* strain TG-1 containing phagemid vector stock was diluted in fresh SB medium containing 100 µg/mL carbenicillin to an optical density at 600 nm (O.D_600_) of 0.1 and incubated at 37 °C with shaking at 250 rpm (Shaking incubator 1585, VWR International). When cell cultures reached an O.D of 0.5 to 0.6, cultures were superinfected with VCSM13 helper phage with a multiplicity of infection (MOI) of 20 and transferred to a 500 mL Erlenmeyer flask containing pre-warmed (37 °C) SB medium and 100 µg/mL carbenicillin. The cultures were kept at 37 °C for 30 min and then shaken at 250 rpm for 30 min at 37 °C. Finally, 50 µg/mL kanamycin were added and continue shaking overnight at 250 rpm at 37 °C. An additional experiment was carried out to evaluate the production of recombinant phage particles at different temperatures of the overnight culture (18 °C and 30 °C), following the protocol previously described. Bacteriophage particles were recovered from the culture supernatant using the PEG/NaCl precipitation method.

### 2.6. Purification of Phage Preparations by PEG/NaCl Precipitation Method

Phage sample purification was carried out by the PEG/NaCl precipitation method [19]. Briefly, purified recombinant phage stocks were prepared by adding 40 g L^−1^ PEG 8000 and 30 g L^−1^ NaCl to 200 mL of the clarified supernatant and briefly mixed in a 500 mL Erlenmeyer flask with slight agitation. The solution was incubated at 4 °C for 2 h for phage precipitation and dispensed into fresh tubes. The phage pellet was generated using a centrifugation step at 10,400× *g* for 40 min (C0650 rotor, Beckman, Brea, CA, USA). The supernatant was discarded, and the pellet was resuspended in 2 mL PBS (2.7 mM KCl, 137 mM NaCl, 10 mM Na_2_HPO_4_, and 1.8 mM KH_2_PO_4_, Desarrollo de Especialidades Químicas, Monterrey, NL, Mexico), pH 7.2. The suspension was transferred to an Eppendorf tube and centrifuged (5417R, Eppendorf, Hamburg, Germany) for 10 min at 16,000× *g* to remove any remaining cell debris. A subsequent second phage precipitation was carried out as previously described. Phage particles were filtered using a sterile 0.2 µm pore size filter, and the phage stock was stored at 4 °C for further analysis. Phage titer was determined by plaque-forming assay and spectrophotometrically by the formula: PFU/mL = ((Abs_269_ − Abs_320_) × (6 × 10^16^))/vector size (bp). For the quantification of recombinant phage particles, the estimation of phage was carried out spectrophotometrically as the infectivity of the phage does not play a role in the phage-based vaccine activity assays, where the relevant aspect is the total number of particles.

### 2.7. Bacteriophage Quantification by Plaque-Forming Assay

Bacteriophage concentration was determined by the plaque-forming assay method [14,23]. Plaque-forming assay was exclusively used for the VCSM13 phage preparation experimental stages (preparation of recombinant phage particles) as in such stage infectivity play a key role in the outcome. Serial dilutions of the phage preparation in SB medium were prepared. Each dilution (1 µL) was dispensed into a sterile graduated microcentrifuge tube containing 50 µL mid-log phase *E. coli* ER2738 culture and incubated by 15 min at 25 °C for phage infection. The culture was mixed with 3 mL of LB top agar (2.5% LB broth, 0.7% agar) which was melted and cooled at 45 °C. The mixture was poured onto a pre-warmed LB agar plate. The plates were allowed to cool and incubated for 16 h at 37 °C. Plaques formed were counted in a Q-20 Quebec colony counter (SOLBAT, S.A de C.V, Puebla, México), and the concentration of phage was determined as pfu mL^−1^. All assays were performed in triplicates.

### 2.8. Characterization of the Recombinant Phages

The expression of the Sbm7462 antigen on the surface of the recombinant bacteriophage M13 was confirmed by Phage ELISA and Dot Blot analysis.

#### 2.8.1. Phage ELISA

Phage ELISA was carried out to evaluate the relative display of the antigens on the surface of bacteriophage M13. Briefly, phage preparations were immobilized overnight at 4 °C on Maxisorp plates (Nunc, Thermo Scientific, Waltham, MA, USA) using TBS buffer. Plates were washed with TBS-T (0.1% tween 20) and then blocked with skim milk 5% in TBS for 2 h at room temperature. Then, antigen expression was detected with mouse THE^TM^ His Tag Monoclonal Antibody (HRP) at 0.5 µg/mL using the Pierce 1-Step Ultra TMB Blotting solution (Thermo Scientific, Waltham, MA, USA) as chromogenic substrate. The reaction was stopped with 2 M H_2_SO_4_ solution (Desarrollo de Especialidades Químicas, Monterrey, NL, Mexico). and absorbance was measured at 450 nm. VCSM13 helper phage was used as negative control and TT phage as positive control.

#### 2.8.2. Dot Blotting

Dot blot was carried out by loading 2 µg of protein per dot (protein expression samples) and 1 × 10^11^ PFU per dot for bacteriophage preparations on a PVDF membrane (Amersham Hybond P 0.45 µm PVDF blotting membrane, GE Healthcare, Chicago, IL, USA) using a vacuum system. Washing, blocking, and antibody incubation were performed according to the Pierce™ Fast Western Blot Kit, ECL Substrate (Thermo Scientific). The membranes were probed using mouse THE^TM^ His Tag Monoclonal Antibody [HRP] (Genscript, Piscatway, NJ, USA) at a concentration of 0.5 µg/mL. Immunoreactive dots were detected using the Pierce 1-Step Ultra TMB Blotting solution (Thermo Scientific). VCSM13 helper phage and TT phage were used as negative and positive controls, respectively. Images were captured with a Bio-Rad Universal Hood Gel Doc System (Hercules, CA, USA).

### 2.9. Densitometry Analysis

Dot blots were analyzed by densitometry with the ImageJ software (NIH, Baltimore, MD, USA) using the gel analysis procedure. To determine the quantity of protein per phage unity, a curve of different concentrations of the standard yellow fluorescent protein containing a His-tag was used.

### 2.10. Generation of Bovine MODCs

Bovine monocyte-derived dendritic cells (MODCs) were generated by adapting the protocol reported by Kangethe et al., 2018 [24] with some modifications. Briefly, bovine peripheral blood mononuclear cells (PBMC) were obtained by a density gradient centrifugation over Ficoll^®^ Paque Plus (GE Healthcare). CD14+ cells were isolated from PBMC through magnetic cell separation using human CD14 microbeads (Miltenyi Biotec, Bergisch Gladbach, Germany) according to the manufacturer’s recommendations (yield 5–10% of the total PBMC and a purity of more than 90%). Purified CD14+ monocytes were then cultured at 300,000 cells/mL/well in a complete RPMI medium (RPMI-1640 supplemented with 10% FBS and 1% penicillin–streptomycin) in a 24-well cell culture plate. Recombinant bovine IL-4 (R&D Systems, Minneapolis, MN, USA) and recombinant bovine GM-CSF (MyBioSource, San Diego, CA, USA) at 40 ng/mL were added and cultured for 2 days to differentiate cells to immature MODCs. Mouse anti-bovine CD80 (clone IL-A159, Bio-Rad) and mouse anti-sheep MHC class II (clone 49.1: Cross-reacts with bovine, Bio-Rad) antibodies were used to evaluate MODCs differentiation. MODCs from day 2 were used for the ex vivo evaluation of the vaccine.

### 2.11. Ex Vivo Evaluation of Vaccine Immunogenicity

To evaluate the capacity of the candidate phage-based vaccine to generate an immune response an ex vivo study was performed, using a bovine monocyte-derived dendritic cell-based assay adapted from the one previously described by Kangethe et al., 2018 [24]. MODCs from day 2 were individually cultured and pulsed in the presence of (1) PBS (negative control), (2) dose of 4 × 10^10^ PFU of Sbm7462 expressing-phage (equivalent to 0.24 µg of recombinant Sbm7462 peptide, (3) a dose of 4 × 10^10^ PFU of VCSM13 helper phage (nude phage), (4) 0.24 µg recombinant Bm86 antigen (positive control) and (5) 1 µg/mL lipopolysaccharide (LPS) (MERCK) as positive control. Purified phage preparations were used. The dose of recombinant Bm86 antigen is equivalent to 0.2% of a single dose in cattle. On day 3, PBMCs from the same animal were co-cultured with MODCs at a ratio of 1:10 MODCs to PBMCs. MODCs harvested at day 3 were stained with CD80 and MHC-II markers to evaluate dendritic cell maturation. On day 6, PBMCs were recovered and analyzed for cell proliferation using the nuclear marker Ki-67 (Alexa Fluor 647 anti-human Ki-67 Antibody, BioLegend, San Diego, CA, USA).

### 2.12. Expression and Purification of Recombinant Sbm7462 Antigen

Sbm7462 peptide was cloned from the phagemid vector pComb3x-antigen into the expression plasmid pET28b using primers that encoded the restriction sites NotI and NdeI. Antigens were expressed in *E. coli* BL21 Star (DE3) after induction with 0.8 mM isopropyl β-D-1-thiogalactopyranoside (IPTG) while shaking at 250 rpm for 16 h at 18 °C. Bacterial cells were harvested by centrifugation at 3500× *g* for 10 min and disrupted with lysis solution (0.8% *w*/*v* Octyl β-D-1-thioglucopyranoside, 0.2 mg/mL lysozyme, 0.2 mM phenylmethanesulfonyl fluoride (PMSF), Tris-HCl 25 mM, pH 7.5) at 8 mL/g pellet. The solution was incubated at room temperature with agitation for 20 min. The cell suspension was sonicated to shear chromosomal DNA (3 cycles of 10 s) and centrifuged to separate soluble and insoluble fractions. Recombinant antigens were purified from the insoluble fraction by resuspending them in a denaturing equilibration buffer (50 mM NaH_2_PO_4_, 300 mM NaCl, 20 mM Imidazole, 8 M Urea, pH 7.4) using a His60 Ni Superflow Resin (Clontech Laboratories Inc., Palo Alto, CA, USA). An on-column refolding protocol was performed using a linear gradient from 8 M to 0 M urea [25]. The refolded antigens were eluted with elution buffer (50 mM NaH_2_PO_4_, 300 mM NaCl, 300 mM Imidazole, pH 7.4, Desarrollo de Especialidades Químicas, Monterrey, NL, Mexico). The recombinant antigen concentration was determined with the BCA (bicinchoninic acid assay, Thermo Scientific, Waltham, MA) assay, and the purity of the samples was estimated by a 12.5% SDS-PAGE analysis (Figure S1, see Supplementary Materials).

### 2.13. Extraction of Bm86 Recombinant Antigen

Bm86 recombinant antigen was extracted from the commercial vaccine Bovimune ixovac (water-in-oil emulsion) (Lapisa, Mexico) since the recombinant protein was required to be in an aqueous solution for cell culture experiments. The extraction was performed with benzyl alcohol at a final concentration of 10% *v*/*v*. The suspension was vortexed for 20 min at maximum speed and centrifuged at 16,100× *g* for 10 min to form phase separation. Recombinant antigen was recovered from the aqueous middle layer [26] A band of ~60 kDa corresponding to the Bm86 antigen from a 12.5% SDS-PAGE gel confirmed the presence of the recombinant protein in the aqueous phase (Figure S2, see Supplementary Materials). Amicon Ultra-15 centrifugal devices (10 kDa) (Millipore) were used to concentrate protein and buffer exchange to PBS (3 cycles at 7500× *g* for 24 min at room temperature). Protein concentration was determined by BCA assay, and antigen samples were stored at −20 °C until use. With this method, a recovery yield of 58.78% was achieved.

### 2.14. In Vivo Assay

Mice were randomly assigned into groups of six: (1) Control, (2) VCSM13 helper phage, (3) Bm86-commercial vaccine, and (4) phage-based vaccine. Animals were inoculated subcutaneously (s.c.) at day 0 (left flank side) and day 12 (right flank side). On day 12, before the second inoculation, mice were bled from the lateral saphenous vein to obtain serum samples. On day 25, mice were anesthetized by an intraperitoneal injection of pentobarbital (70 mg/kg), blood was then collected by cardiac puncture.

Mice were then sacrificed by cervical dislocation, and the spleen was collected. Splenocytes were extracted by mechanical disruption. PBMCs from the spleen were obtained by density gradient centrifugation using Ficoll-Paque plus (450× *g* for 30 min at 20 °C with brake turned off). 2 × 10^5^ cells were plated in a 24-well culture plate in complete RPMI medium to a final volume of 200 µL. Then, cells from each group were pulsed with: (1) PBS (negative control), (2) 4 × 10^10^ PFU of Sbm7462 expressing-phage (equivalent to 0.24 µg of recombinant Sbm7462 peptide, (3) 4 × 10^10^ PFU of VCSM13 helper phage (nude phage), (4) 0.24 µg recombinant Bm86 antigen (positive control) and (5) 1 µg/mL lipopolysaccharide (LPS) as positive control during 96 h. Total cell count to evaluate cell proliferation was carried out by the trypan blue staining method.

Serum samples obtained from day 12 and day 25 were used to evaluate antibodies by a dot blot assay.

### 2.15. Antibody Evaluation by Dot Blot Assay

For the evaluation of specific antibodies from the serum of immunized animals, Sbm7462 recombinant antigen (4 µg of protein per dot), VCSM13 helper phage (1 × 10^11^ PFU per dot), and Bm86 recombinant antigen (4 µg of protein per dot) were applied on an individual PVDF membrane using the Bio-dot Microfiltration apparatus (Bio-Rad). Serum samples from all mice were tested for each antigen. Serum samples were diluted 1:100. Washing, blocking, and antibody incubation were performed according to the Pierce™ Fast Western Blot Kit, ECL Substrate (Thermo Scientific). TMB was used as the chromogenic substrate. Dot blots were analyzed as previously described in the dot blotting section.

### 2.16. Special Equipment

BD Accuri C6 flow cytometer (BD Biosciences, Franklin Lakes, NJ, USA) was used for cell viability and cellular markers measurements.

### 2.17. Statistical Analysis

GraphPad Prism Software (San Diego, CA, USA) was used for statistical analysis. All results are presented as mean value ± standard deviation. The Student’s *t*-test was used to evaluate the difference between groups. The unpaired non-parametric Mann Whitney test was applied to compare the treatment groups for the in vivo and ex vivo assays. All *p*-values < 0.05 were considered significant.

## 3. Results

### 3.1. Construction and Characterization of the Recombinant Bacteriophage

**Figure 1:**
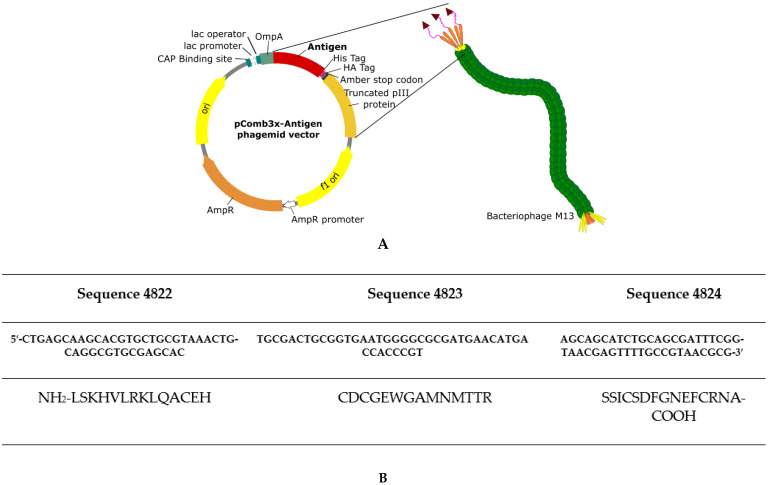
Schematic representation of the phage-based vaccine design. (**A**). A pComb3x phagemid vector was used to fuse antigens to a truncated pIII protein of bacteriophage M13. It contains a His-tag at the carboxy-terminal to the antigen fragment for IMAC purification. A hemagglutinin (HA) tag was also inserted to facilitate detection using an anti-HA antibody. The amber stop codon allows the expression of soluble antigens in non-suppressor strains of bacteria without eliminating the truncated pIII gene fragment [27]. (**B**). Sequence of the Sbm7462 antigen. Peptides derived from the Bm86 protein are depicted [28]. The peptides are continuously aligned in one expression construct.

Figure 1A shows a schematic view of the phagemid vector used for construction of recombinant bacteriophage.

A recombinant phage-displaying the epitopes of Bm86 protein [21] was developed. Sbm7462 antigen is formed by the continuous fusion of three inferred immunogenic epitopes of Bm86 protein: peptide sequences 4822 (a.a. 398–411), 4823 (a.a. 21–35) and 4824 (a.a. residues 132–145) (Figure 1B).

**Figure 2:**
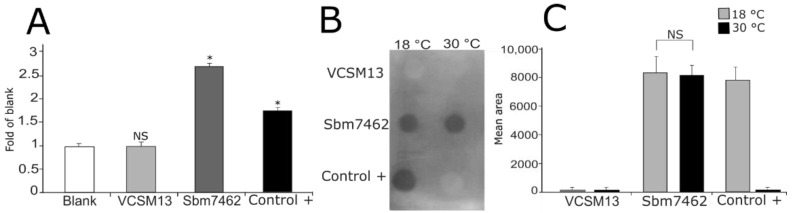
Phage characterization. Expression of recombinant antigens on the surface of bacteriophage M13. (**A**) Phage ELISA. Recombinant phage expressing Sbm7462 antigen was probed in a direct ELISA assay using anti-His antibodies. (**B**) Dot blots of bacteriophages expressing antigens. Expression of antigens on phage surface at different temperatures of a 17 h culture (18 °C, 30 °C). VCSM13 phage as negative control for phage samples (1 × 10^11^ PFU per dot). SBm7462 phage-based vaccine at 1 × 10^11^ plaque-forming units (PFU) per dot. Sbm7462 represents the antigen displayed on phage. Control + is represented by a phage expressing a tetanus toxin Fab produced through the pComb3x phagemid vector. Samples were probed using mouse anti-His-Tag. (**C**) Densitometric analysis of the dot blots. * *p*-value < 0.05, NS—not significant.

To evaluate the expression of proteins on the surface of bacteriophage M13, the phagemid vector allows the expression of a His-Tag and an HA-Tag between the protein to be displayed and the truncated pIII protein (Figure 1). These tags facilitate the detection of the displayed protein by using anti-His or anti-HA antibodies. To confirm the expression of antigen Sbm7462 on the surface of phage particles, an ELISA assay and dot blotting analysis were carried out using the antibodies previously mentioned. As shown in Figure 2A, antigen was correctly expressed as a fusion of the truncated pIII protein. Recombinant phages were assayed with a His-Tag antibody, and a 2.73-fold increase was found compared to control (blank). In addition, there was no significant difference in VCSM13 (negative control phage) compared to the blank, demonstrating that nude phage comprises a suitable phage negative control.

Phage production was initially performed at 37 °C. Unfortunately, there was no protein expression at that temperature. Based on this result, phage production was carried out at 18 °C and 30 °C. As seen in Figure 2B, Sbm7462 antigen was adequately expressed at both temperatures. These results indicate that temperature plays an essential role in the expression of proteins and peptides on the surface of the bacteriophage M13.

In agreement with the ELISA assay, the VCSM13 phage did not show any signal in the dot blot assay. Densitometry analysis of dot blotting (Figure 2C) showed no significant difference between both temperatures tested for the expression of Sbm7462 peptide. Furthermore, antigen expression was similar to that found by the display of tetanus toxin Fab (control+), demonstrating that the Bm86-derived antigen is suitable for being displayed at the surface of bacteriophage M13.

**Figure 3:**
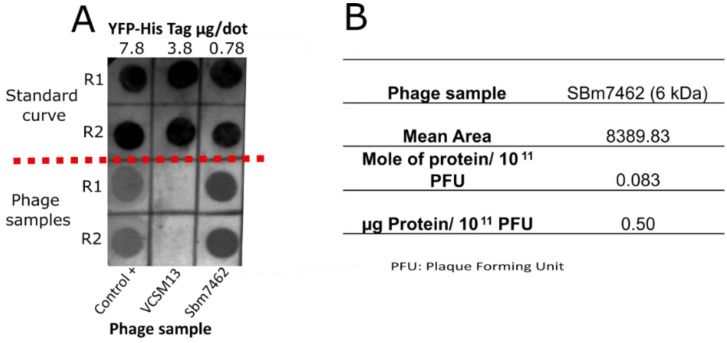
Determination of antigen expression on the surface of recombinant phages. (**A**) Curve of mean area vs mole of recombinant yellow fluorescent protein (YFP) that contains a His-tag was used to determine by densitometry analysis the mole of antigen expressed per PFU unit. Different quantities of YFP were applied in duplicate. Different concentrations of yellow fluorescent protein were used to construct a standard curve for densitometry analysis. For phage samples, 1 × 10^11^ PFU per dot were applied. (**B**) Determination of mole and µg of protein (Sbm7462 antigen) per phage unit (PFU). R, replicate. Control +, phage expressing a tetanus toxin Fab, VCSM13, negative control phage. Sbm7462, phage-based vaccine (Antigen Sbm7462 displayed on phage).

Once the antigen expression on the phage surface was confirmed, it was decided to determine the quantity of recombinant protein displayed per phage unit to determine the doses of the phage-based vaccine for the immune response assays with higher accuracy. To determine the quantity of antigen expressed per phage unit, different quantities of a recombinant yellow fluorescent protein (YFP) were used in the dot blot (Figure 3A). Since the YFP contains a His-tag, the µg of antigen displayed per PFU was calculated through the YFP standard curve (µg of protein vs. mean area of the densitometry analysis). As shown in Figure 3B, Sbm7462 antigen was expressed in 0.5 µg of protein per 1 × 10^11^ PFU. This data was then used for the ex vivo and the in vivo experiments. Notably, the relevance of this determination relies on the importance of the dose of antigen in the immune response assays.

### 3.2. Phage-Based Vaccine Induces Maturation of Bovine Monocyte-Derived Dendritic Cells

**Figure 4:**
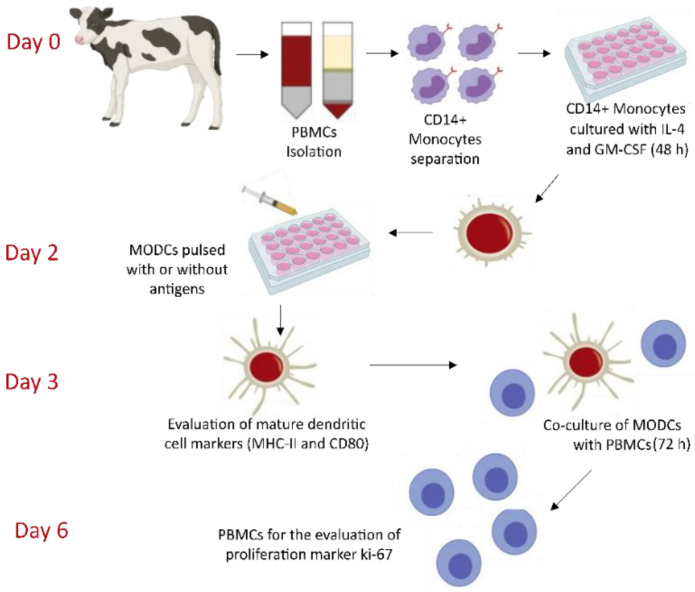
Illustration of the evaluation of the immune response of the phage-based vaccine using the bovine MODC-based assay. On day 0, CD14+ monocytes were isolated from PBMCs. Purified CD14+ monocytes were cultured in the presence of bovine IL-4 and GM-CSF. On day 2, cultured monocytes were evaluated for the presence of dendritic cell differentiation markers (CD80 and MHC-II). Once the presence of differentiation markers was confirmed, the cells were pulsed with or without the antigens. On day 3, freshly prepared PBMCs from the same animal were co-cultured with the antigen-treated dendritic cells. On day 6, PBMCs were harvested and evaluated for the proliferation of lymphocytes by measuring the expression of the ki-67 protein.

After the recombinant phage was characterized and the quantity of antigen per phage unit was determined, it was decided to evaluate the immune response of the phage-based vaccine in a dendritic cell-based ex vivo assay (Figure 4). A schematic representation of the main steps involved in evaluating the vaccine immunogenicity ex vivo is described in Figure 4. Remarkably, this kind of ex vivo assays has been successfully developed and used to evaluate vaccine immunogenicity [24,29]. Although in vivo assays represent the standard model to measure vaccine immunogenicity, the ex vivo assays as the one used in the present research work are good models that can precede in vivo assays. This can minimize the number of animals used for the vaccine evaluation, increasing the number of vaccine treatments tested.

**Figure 5:**
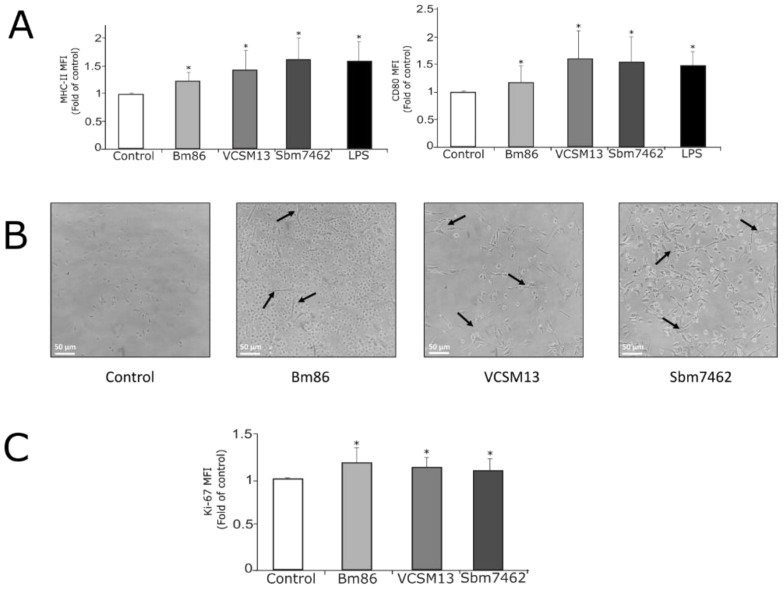
Phage-based vaccine induces maturation of bovine monocyte-derived dendritic cells. (**A**) Representative graphs showing the fold increase of mean fluorescence intensity (MFI) of MHC class II and CD80+ of bovine MODCs untreated (negative control), pulsed with 0.24 µg Bm86 antigen (positive control), 4 × 10^10^ PFU of VCSM13 helper phage (nude phage), 4 × 10^10^ PFU of Sbm7462 expressing-phage (equivalent to 0.24 µg of recombinant Sbm7462 peptide) or stimulated with 1 µg/mL of LPS (positive control) during 18.5 h. Each well contained 300,000 cells. (**B**) Morphology of monocyte-derived dendritic cells pulsed with or without antigens. (**C**) Proliferation of PBMC restimulated with or without antigens measured through the expression of protein ki-67. Graphs shown represent the means (±SD) of six independent experiments. *n* = 6. * *p*-value < 0.05 compared to control group.

As shown in Figure 5A, MODCs pulsed with the phage-based vaccine showed a significant fold increase in dendritic cell maturation markers CD80 (1.5-fold of MFI) and MHC-II (1.6-fold of MFI) when compared to unstimulated cells (control group). These findings resemble those seen in cells stimulated with the positive control (LPS), which showed a fold increase of 1.2 and 1.4 for CD80 and MHC-II, respectively. VCSM13 phage also showed a significant increase in both maturation markers, 1.4-fold for CD80 and 1.6-fold for MHC-II.

MODCs maturation was also confirmed by the representative DC-like morphology (projection of dendrites) presented in cells after being pulsed with antigens (Figure 5B). As expected, MODC’s morphology in the VCSM13 and phage-based vaccine treatments was very similar (remarked with arrows in Figure 5B). The ability of VCSM13 phage to generate maturation of dendritic cells confirms that the phage particle itself is capable of being taken up by dendritic cells and induce their maturation. In addition, MODCs stimulated with the recombinant antigen Bm86 show a remarkable DC-like morphology. Unstimulated cells did not develop the morphology of mature dendritic cells. Thus, all these results confirm that phage-based vaccine-induced bovine MODCs maturation.

### 3.3. Bovine MODCs Trigger Specific Priming of PBMCs

Once the maturation of dendritic cells was confirmed, it was decided to evaluate the ability of bovine MODCs to prime PBMCs. Cell proliferation was measured by the expression of Ki-67 protein, a proliferation marker [30], in PBMCs co-cultured with antigen-stimulated dendritic cells. As shown in Figure 5C, the proliferation of PBMCs occurred in all antigen-stimulated groups. The control group did not show signs of proliferation through the evaluation of Ki-67 protein. These results indicate that the phage-based vaccine induces the generation of MODCs capable of efficiently priming antigen-specific mononuclear cells in the ex vivo assay.

### 3.4. Vaccination of Balb/c Mice with the Phage-Based Vaccine Induces Spleen’s PBMC Proliferation and Generates Specific Antibody Production

**Figure 6:**
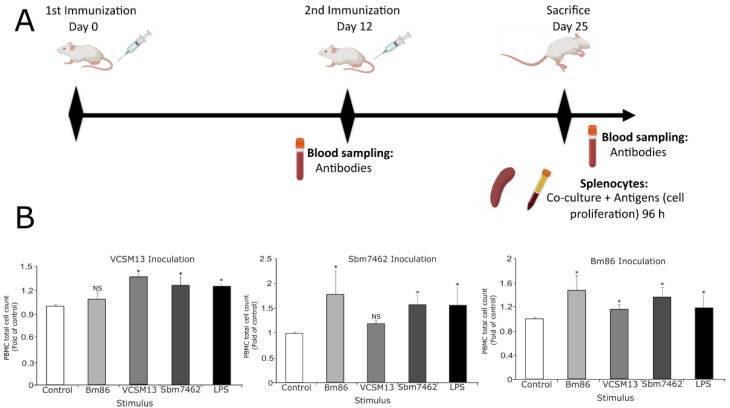
Evaluation of the immune response of the phage-based vaccine in vivo. (**A**). Schematic illustration of the phage immunization and serum sampling schedule. Mice were inoculated subcutaneously with PBS (control group), 1 × 10^12^ PFU VCSM13 (control phage), 5 µg commercial antigen BM86, and 1 × 10^12^ PFU Sbm7462-phage vaccine equivalent to 5 µg antigen. All treatments were administered in 100 µL of PBS. (**B**). PBMCs obtained from spleen at day 25 were pulsed with (1) PBS (negative control), (2) 4 × 10^10^ PFU of Sbm7462 expressing-phage (equivalent to 0.24 µg of recombinant Sbm7462 peptide, (3) 4 × 10^10^ PFU of VCSM13 helper phage (nude phage), (4) 0.24 µg recombinant Bm86 antigen (positive control) and (5) 1 µg/mL lipopolysaccharide (LPS) as positive control. Sbm7462 represent the antigen Sbm7462 displayed on phage. Total cell count was performed after 96 h. *n* = 6. * *p*-value < 0.05 compared to control group, NS—not significant.

To evaluate the immune response of the phage-based vaccine, we performed a vaccination model of Balb/c mice. Figure 6A shows the general steps followed to perform the in vivo assay. To investigate if splenocytes from vaccinated mice could proliferate after being restimulated, cells obtained from each immunized group were individually cultured in the presence of PBS, VCSM13, phage-based vaccine, Bm86 antigen, or LPS. As shown in Figure 6B, cells obtained from VCSM13 vaccinated mice show a significant increase in total cell count when restimulated with the specific antigen VCSM13, also with Sbm7462 and LPS. Whereas cells obtained from Sbm7462-vaccinated mice show a significative increase when restimulated with the specific antigen Sbm7462, Bm86 and LPS. Finally, cells from Bm86-vaccinated mice increased in cell count with all stimuli. These results demonstrate the capability of phage Sbm7462 to respond to Bm86 antigen since the Sbm7462 peptide is derived from the Bm86 protein. The same result was obtained when cells from the Bm86 group were stimulated with the recombinant antigen Sbm7462.

**Figure 7:**
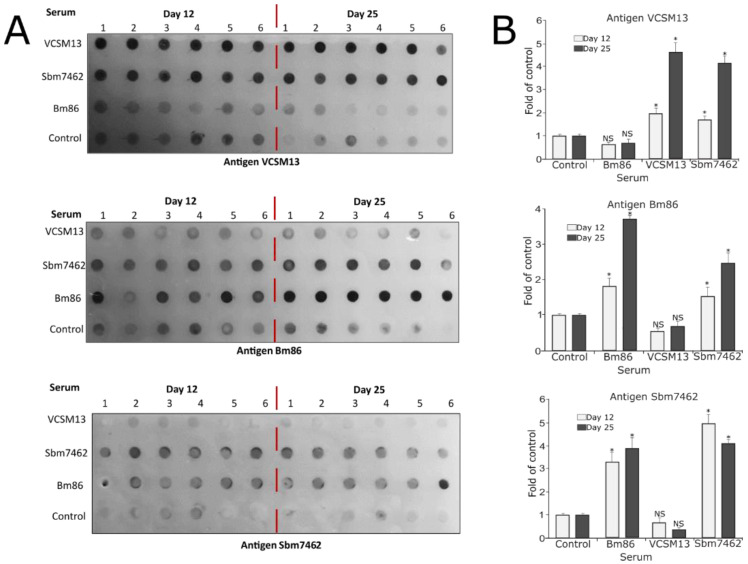
Antibody response. (**A**). Dot blots of the antibody evaluation from mice serum obtained by day 12 and day 25. Serum samples from all mice were tested with individual membranes coated with the respective antigens. Mice were inoculated subcutaneously with PBS (control group), 1 × 10^12^ PFU VCSM13 (control phage), 5 µg commercial antigen BM86, and 1 × 10^12^ PFU Sbm7462-phage vaccine equivalent to 5 µg antigen. All treatments were administered in 100 µL of PBS. (**B**). Densitometry analysis of the dot blots. *n* = 6. * *p*-value < 0.05 compared to control group, NS—not significant.

After demonstrating that the specific restimulation of cells from vaccinated mice could induce a specific response, the antibodies in the serum of the treated mice were evaluated. As shown in Figure 7A,B, serum from mice immunized with the VCSM13 control phage and the Sbm7462 phage-based vaccine showed a significative specific antibody response against antigen VCSM13. Serum from mice vaccinated with the commercial Bm86 antigen and the Sbm7462-expressing phage showed a significant specific antibody response against Bm86 and Sbm7462 antigens. These observations agree with the results observed in the restimulation of PBMCs from spleen. Interestingly, mice immunized with VCSM13 phage also generated an antibody response to phage proteins. As seen in Figure 7A,B, the antibody production was higher at day 25 than at day 12 for VCSM13 and Bm86-treated mice. These data confirms that the phage-based vaccine is capable to generate a specific antibody response to the antigen displayed in the phage particle.

## 4. Discussion

Cattle tick represents a major veterinarian problem in Mexico and worldwide [2,9,10]. Although several strategies intended to solve this problem have been developed, none of them are entirely effective. In this research, we have developed and characterized a candidate vaccine against this ectoparasite. In the present research work, we focused on developing and assessing the potential of a phage-based vaccine against cattle tick.

The expression of antigens on the surface of phage particles has several advantages compared with formulations of individual recombinant antigens or synthetic peptide vaccines [19]. Phage-based vaccines are cost-effective since recombinant phages can be effectively produced in huge quantities from an infected bacterial culture [14,31]. Since the bacteriophage M13 is secreted into the medium during the fermentation process, the downstream processing is straightforward and cheap for this kind of bioproduct. Moreover, bacteriophages are resistant to several stresses, which confers stability to both, the viral particle and antigens fused to the protein on their surface. Notably, it has been reported that bacteriophage particles can trigger an immune response on their own [32]. This prominent characteristic allows the use of phage particles as antigen delivery vectors without the need of adjuvants in the formulation. These essential characteristics of bacteriophages are helpful for the development of novel, stable, and more cost-effective vaccines.

As discussed above, our research group developed a candidate vaccine against cattle tick expressing an antigen on the surface of bacteriophage M13. In this work, we used a phagemid vector to display the antigens fused to the pIII protein located on the surface of the M13 phage. Through immunodetection techniques such as ELISA and dot blot, we effectively identified the expression of the antigen. Although the pIII protein is only expressed three to five times on each phage particle, the considerable amount of phage particles in each plaque-forming unit is sufficient to induce a clear response with the immunodetection strategies used here. Expression of antigens fused to the major coat protein of M13 phage could represent an excellent strategy to increase the number of antigen molecules displayed per each viral particle. This strategy has been previously reported for proteins with a maximum molecular weight of 7 kDa. Fortunately, the size of the Sbm7462 antigen (6 kDa) is in the range for proper expression on the major coat protein of bacteriophage M13. Moreover, the Sbm7462 peptide, which consists of the continuous fusion of three predicted immunogenic B and T cell epitopes of Bm86 protein (43 amino acids), has shown protection and high efficacy against cattle tick [11]. Furthermore, the sequences of the epitopes of Sbm7462 peptide have been found to be conserved in several *R. microplus* strains [28]. The size of the protein is a relevant feature to consider when it is intended to be displayed on the surface proteins of the phage. Nowadays, the usage of bioinformatic tools to identify epitopes of antigens allows the design and display of practically any desired antigen. Another strategy that could be helpful to increase the relative expression of antigens fused to the pIII protein is the modification of the signal peptide of the phagemid vector. This strategy was previously described by Steiner et al., 2006 [33]; the authors directed the protein expression through the signal recognition particle (SRP) translocation system. In this sense, the expression of several proteins was increased when compared to the post-translational Sec translocation system. The pComb3x phagemid vector used in this work contains the OmpA signal peptide that belongs to the Sec pathway.

In vivo assays are the gold standard for the evaluation of vaccine immunogenicity [24]. However, several studies have reported applying ex vivo assays based on dendritic cells to measure vaccine immunogenicity, thus reducing the number of animals utilized to evaluate different treatments of candidate vaccines [24,34]. Based on this, it was decided to use an ex vivo bovine dendritic cell-based assay to evaluate the immune response of the developed candidate bacteriophage-based vaccine. It was found that the phage that displayed the Sbm7462 antigen was able to induce MODCs maturation (increase of MHC-II and CD80) at the same level of the commercial antigen Bm86 and the positive control LPS. In addition, VSCM13 phage induced MODCs maturation, indicating that the native phage (without antigens on their surface) can generate an initial immune response, validating the ability of bacteriophage M13 to act as adjuvant and promote an immune response. Several authors have reported this capability of filamentous phages for the development of phage-based vaccines [35,36]. Furthermore, this is the first time a phage-based vaccine has been evaluated in an ex vivo model. The results obtained in this research are comparable to those obtained in previous studies using bovine DC-based ex vivo assays evaluating other types of vaccines (inactivated) [24]. These results support that phage particles represent a suitable platform for developing vaccines for use in the veterinary industry.

The adjuvant capacity of bacteriophage M13 has several advantages in the development of a vaccine, primarily due to the reduction of formulation costs and, importantly, the development of a proper immune response [31,37,38]. Although the innate structure of bacteriophage M13 (CpG motifs, repeated protein structure) confers the adjuvant capacity of filamentous phages, it is relevant to consider that residual LPS also plays a crucial role in the development of the immune response [37]. It has been reported that the complete elimination of LPS from the phage preparations did not change the immune response compared to phage preparations containing residual LPS [38]. Although the presence of LPS on phage preparations has been found not to influence the effect of phage particles, it has also been reported that the LPS linked to the phage particles may participate in the immunogenicity of filamentous phages [38]. In this work, phage particles were double precipitated, achieving an effective removal of LPS. Nevertheless, the presence of residual LPS may have contributed to the immune response found here. Considering that LPS is a prototype of pathogen-associated molecular patterns (PAMPs), the contribution to phage’s immunogenicity could be attributed to that.

Although the ex vivo assay represents a general view of the responses associated with the vaccine, a bovine in vivo assay is still necessary to confirm the vaccine’s effectiveness. However, bovine in vivo models present several technical and time-dependent difficulties. For this reason, we decided to perform a murine in vivo assay to obtain a better view of the immune response, as murine models can be helpful for the study of mechanisms of immune protection and determine the safety and efficacy of vaccines [39]. Our results demonstrated that the phage-based vaccine could generate specific antibodies to the antigen fused to the phage particle. This important finding suggests that our candidate phage-based vaccine could produce antibody response in bovines. A previous report stated that humoral immune response was important in the mechanism of action of Bm86 and Sbm7462 antigens when they were inoculated in bovines [11]. Interestingly, it is essential to note that the phage that displayed the Sbm7462 antigen induced the production of antibodies that recognized the Bm86 commercial protein. This could be explained since the Sbm7462 peptide is derived from the Bm86 protein [28]. In the same way, mice inoculated with the commercial Bm86-based vaccine generated antibodies that were detected with the recombinant Sbm7462 antigen. From the above, we hypothesize that our candidate vaccine could be effective in protecting cattle against ticks. However, further characterization of the protective effects of the antibody response in bovines is necessary to demonstrate the efficacy of our candidate phage-based vaccine. Moreover, the cytokine profile should be studied to confirm the type of immune response. The study of other immunogenic peptides derived from well-characterized antigens such as subolesin antigen would be very interesting to develop a multi-epitope phage-based vaccine.

Regarding the immunological capacity of the candidate vaccine, we demonstrated that it elicited the proliferation of lymphocytes involved in cellular and humoral responses. The immune response associated with the vaccine could be directly related to the engulfment of phage particles by presenting cells of the immune system, such as the monocyte-derived dendritic cells used in the present study [35].

The results in the present research work demonstrate that the designed phage-based vaccine was capable of inducing bovine MODCs maturation and proliferation of bovine PBMCs. Specific antibody production was confirmed with the in vivo assay. PBMCs obtained from the spleen of vaccinated mice were capable to proliferate when pulsed with specific antigens. The candidate phage-based vaccine presented in this research can generate a specific immune response in ex vivo and in vivo assays. Thus, the candidate phage-based vaccine represents a potential antigen delivery system for the control of cattle ticks. Since this platform allows the display of any antigen, it could be used for the development of novel vaccines for human and veterinary applications.

In brief, the present research demonstrates that phage display technology can be applied to develop novel and potent antigen delivery systems. The evaluation through an in vivo assay with bovine animals is necessary to validate our candidate vaccine’s capacity to induce protective immunological responses against cattle tick. Our platform represents a promising strategy in generating novel, stable and cost-effective vaccines in the veterinary field for several diseases.

## 5. Conclusions

Our results demonstrated that the candidate phage-based vaccine developed in this research work can induce both humoral and cellular immune responses when evaluated in a bovine-derived dendritic cell-based ex vivo assay. Moreover, this is the first study reporting the immunogenicity of a phage-based vaccine evaluated in a bovine-derived dendritic cell-based ex vivo assay. Remarkably, the candidate vaccine showed a similar immune response to the available commercial Bm86-based vaccine. Further studies of the phage-based vaccine in a bovine in vivo assay are needed to validate the immune protective capacity of the developed vaccine against cattle tick. This platform can also be employed to develop potent and novel antigen delivery systems for several veterinary diseases.

## Data Availability

The data presented in this study are available on request from the corresponding author.

## Supplementary Materials

The following are available online at https://www.mdpi.com/article/10.3390/pharmaceutics13122018/s1, Figure S1: SDS-PAGE analysis of the expression of recombinant antigens. (1) Purified recombinant Sbm7462 antigen; (2) Elution 2 Subolesin; (3) Elution 3 Subolesin; (4) Elution 5 Subolesin; (5) No binding Subolesin; (6) Elution 8 Subolesin; (7) Molecular weight marker, Figure S2: SDS-PAGE analysis of the extraction of the recombinant antigen Bm86 from the commercial vaccine Bovimune Ixovac. (1) Bm86 antigen from bottom phase; (2–4) Bm86 antigen from middle phase obtained with independent experiments; (5) Molecular weight marker.
