# Supplementary material for: Evaluation of the Immune Response of a Candidate Phage-Based Vaccine against Rhipicephalus microplus (Cattle Tick)
Source: Pharmaceutics. 2021 Nov 26;13(12):2018. doi: 10.3390/pharmaceutics13122018 (PMC8706106; doi:10.3390/pharmaceutics13122018)
Supplement: Supplementary file 1 [file pharmaceutics-13-02018-s001.zip › SM/pharmaceutics-1419957-supplementary-update.pdf]

pharmaceutics

# Supplementary Materials: Evaluation of the Immune Response of a Candidate Phage-Based Vaccine against *Rhipicephalus microplus* (Cattle Tick)

**Alejandro González-Mora, Kenny Misael Calvillo-Rodríguez, Jesús Hernández-Pérez, Marco Rito-Palomares, Ana Carolina Martínez-Torres and Jorge Benavides**

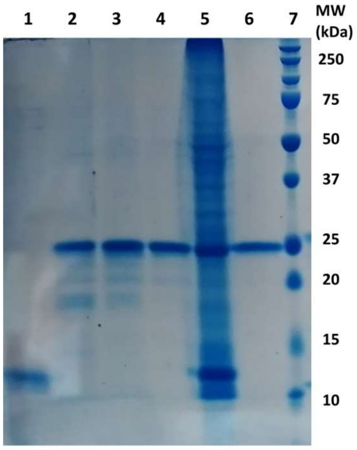


**Figure S1.** SDS-PAGE analysis of the expression of recombinant antigens. (**1**) Purified recombinant Sbm7462 antigen; (**2**) Elution 2 Subolesin; (**3**) Elution 3 Subolesin; (**4**) Elution 5 Subolesin; (**5**) No binding Subolesin; (**6**) Elution 8 Subolesin; (**7**) Molecular weight marker.

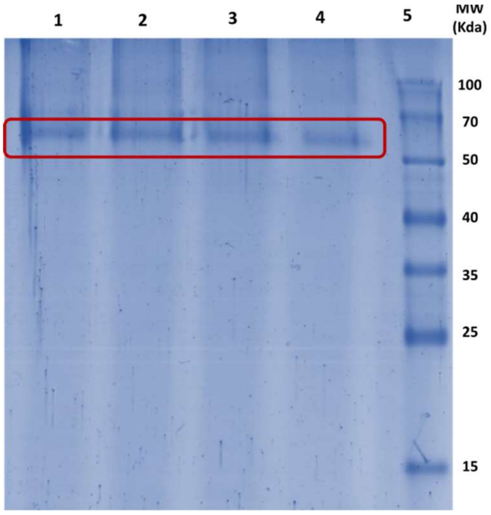


**Figure S2.** SDS-PAGE analysis of the extraction of the recombinant antigen Bm86 from the commercial vaccine Bovimune Ixovac. (**1**) Bm86 antigen from bottom phase; (**2-4**) Bm86 antigen from middle phase obtained with independent experiments; (**5**) Molecular weight marker.
